# The first oxazoline adduct of Zn(acac)_2_: bis­(acetyl­acetonato-κ^2^ *O*,*O*′)(2-phenyl-2-oxazoline-κ*N*)zinc(II)

**DOI:** 10.1107/S1600536808042712

**Published:** 2008-12-20

**Authors:** Ignacio del Río, Robert A. Gossage

**Affiliations:** a Departamento de Química Orgánica e Inorgánica, Facultad de Química, Universidad de Oviedo, Avda. Julián Clavería, 8, 33006 Oviedo, Spain; b Department of Chemistry & Biology, Ryerson University, 350 Victoria Street, Toronto, ON M5B 2K3, Canada

## Abstract

The title material, [Zn(C_5_H_7_O_2_)_2_(C_9_H_9_NO)], was synthesized by the treatment of bis­(acetyl­acetonato)zinc(II) monohydrate with 2-phenyl-2-oxazoline. The Zn atom is coordinated by two chelating acetyl­acetonate groups and one oxazoline ligand in the apical position of a slightly distorted square-pyramidal metal–ligand geometry.

## Related literature

For general background, see: Addison *et al.* (1984 ▶); Itoh *et al.* (1989 ▶); Kaeriyama (1974 ▶); Williams (1989 ▶). For related structures, see: Barclay *et al.* (2003 ▶); Brahma *et al.* (2008 ▶); Decken *et al.* (2006 ▶); Fronczek *et al.* (1990 ▶); Gossage & Jenkins (2008 ▶); Gossage *et al.* (2009 ▶); Hamid *et al.* (2005 ▶); Qian *et al.* (2006 ▶).
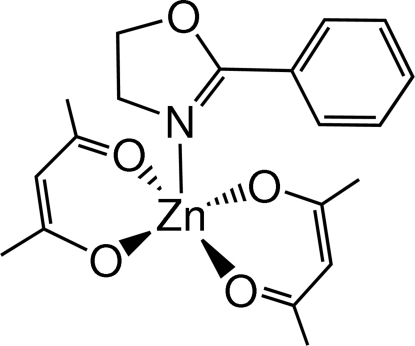


## Experimental

### 

#### Crystal data

[Zn(C_5_H_7_O_2_)_2_(C_9_H_9_NO)]
*M* *_r_* = 410.77
Orthorhombic, 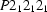

*a* = 9.5009 (3) Å
*b* = 14.1674 (4) Å
*c* = 14.2407 (5) Å
*V* = 1916.84 (11) Å^3^
*Z* = 4
Cu *K*α radiation
μ = 2.03 mm^−1^
*T* = 200 (2) K
0.28 × 0.15 × 0.08 mm

#### Data collection

Nonius KappaCCD diffractometer
Absorption correction: refined from Δ*F* (Parkin *et al.*, 1995 ▶) *T*_min_ = 0.542, *T*_max_ = 0.859
8596 measured reflections
3600 independent reflections
3466 reflections with *I* > 2σ(*I*)
*R*_int_ = 0.035

#### Refinement

*R*[*F* ^2^ > 2σ(*F* ^2^)] = 0.030
*wR*(*F* ^2^) = 0.080
*S* = 1.05
3600 reflections
236 parameters
H-atom parameters constrained
Δρ_max_ = 0.24 e Å^−3^
Δρ_min_ = −0.29 e Å^−3^

### 

Data collection: *COLLECT* (Nonius, 1998 ▶); cell refinement: *SCALEPACK* (Otwinowski & Minor, 1997 ▶); data reduction: *SCALEPACK* and *DENZO* (Otwinowski & Minor, 1997 ▶); program(s) used to solve structure: *DIRDIF96* (Beurskens *et al.*, 1996 ▶); program(s) used to refine structure: *SHELXL97* (Sheldrick, 2008 ▶); molecular graphics: *EUCLID* (Spek, 1982 ▶); software used to prepare material for publication: *SHELXL97*.

## Supplementary Material

Crystal structure: contains datablocks I, global. DOI: 10.1107/S1600536808042712/kj2105sup1.cif

Structure factors: contains datablocks I. DOI: 10.1107/S1600536808042712/kj2105Isup2.hkl

Additional supplementary materials:  crystallographic information; 3D view; checkCIF report

## Figures and Tables

**Table 1:** Selected bond lengths (Å)

N1—Zn1	2.0844 (19)
O2—Zn1	2.0253 (19)
O3—Zn1	2.0136 (17)
O4—Zn1	2.0359 (19)
O5—Zn1	2.0169 (18)

**Fig. 1.**
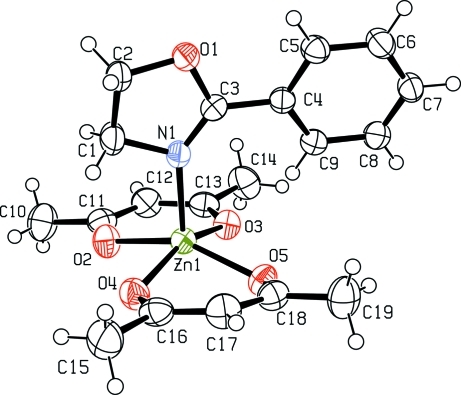
View of the title compound. Ellipsoids are drawn at the 30% probability level.

## supplementary crystallographic information

### Comment

In recent years, zinc acetylacetonate (ZAA) complexes have found significant applications in CVD technology (Itoh *et al.*, 1989; Kaeriyama, 1974; Williams, 1989), as Lewis acids in coordination chemistry (Brahma *et al.*, 2008; Fronczek *et al.*, 1990; Hamid *et al.*, 2005) and are used for the formation of inorganic polymers (Qian *et al.*, 2006). In many cases, the ZAA species are paired with *N*-donor ligands to modify and tune their physical and spectroscopic properties. We recently disclosed the syntheses and structural characterization of a number of Zn coordination compounds containing monodentate 2-oxazoline ligands (Barclay *et al.*, 2003; Decken *et al.*, 2006; Gossage & Jenkins, 2008; Gossage *et al.*, 2009). These materials represent a number of structural motifs around the Zn^2+^coordination sphere which includes the observation of pseudo-tetrahedral and distorted trigonal bipyramidal geometries. The nature of these coordination motifs are obviously influenced by both the nature of the oxazoline ligand(*s*) and the structure and donor properties of the various formal anions appending the Zn atom. In this report, we expand these investigations to include ZAA precursors and describe our first results in the coupling of oxazolines to a ZAA unit.

The crystal structure determination of the title compound reveals that the central Zn atom is coordinated by four *O*-atoms of two chelating (*i.e*.,κ^2^*O,O'*) acetylacetonato (acac) fragments in addition to the attachment of the oxazoline ligand *via* the *N*-atom. Th Zn–N bond length is similar to that of the distorted tetrahedral (at Zn) complexes (Barclay *et al.*, 2003) [Zn*X*_2_(Phox-κ^1^*N*)_2_] (Zn–N = 2.026 (2) and 2.050 (2) Å for *X* = Cl; Zn–N = 2.025 (3) and 2.053 (3) Å for *X* = Br; Phox = 2-phenyl-2-oxazoline) and the related (Decken *et al.*, 2006) five-coordinate species [Zn(S_2_CNEt_2_-κ^2^*S,S'*)_2_(Phox-κ^1^*N*)] (Zn–N = 2.082 (4) Å). The Zn–O bond lengths of the formally anionic acac ligands of the title material are all inequivalent but fall within a narrow range (2.01–2.04 Å).

The Zn-phox complex reported by Decken *et al.* (2006) possesses a coordination motif around Zn that is best described (Addison *et al.*, 1984) as highly distorted trigonal bipyramidal (τ = 0.65) whereas the title complex is strongly disposed towards a structure of idealized square pyramidal (τ = 0.04). The τ parameter is a numerical descriptor defined as unity for pure trigonal bipyramidal structures and zero for true square pyramidal ones (Addison *et al.*, 1984).

This coordination geometry places the *N*-bound oxazoline in the formal apical position of such a square pyramid. ZAA complexes containing *N*-donor lignads are often found to be octahedral in nature with an "O_4_N_2_" donor atom set (Brahma *et al.*, 2008; Fronczek *et al.*, 1990; Hamid *et al.*, 2005; Qian *et al.*, 2006). The title material therefore represents the more rare "O_4_N"-type compound.

Our structural studies have so far observed both four- and five-coordinate Zn*X*_2 _(*X* = halide, S_2_CNRR', acac) oxazoline systems (Barclay *et al.*, 2003; Decken *et al.*, 2006; Gossage & Jenkins, 2008; Gossage *et al.*, 2009). Intriguingly, an octahedral Zn-oxazoline complex has yet to be observed; this suggests that perhaps the use of weakly coordinating anions (*e.g*., NO_3_^-^, ClO_4_^-^,*etc*.) and/or sterically smaller oxazolines may assist us in discovering this structural class of Zn materials. Our future work will involve such investigations.

### Experimental

The treatment of a benzene solution of commercially available bis(acetylacetonato-κ^2^*O*,*O'*)zinc(II) (in the form of the monohydrate) with an excess of Phox leads to the formation of a clear and colourless solution. The removal of volatile components (*vacuo*) followed by re-crystallization (CH_2_Cl_2_/Et_2_O) of the resulting off white oily solid leads to the isolation of colourless crystals of the *product* (65%).

### Refinement

All the hydrogen atom positions were calculated and refined riding on their parent atoms with C—H = 0.96 Å (methyl), 0.97 Å (methylene) or 0.93 Å (aromatic) with U_iso_(H) = 1.5U_eq_ (methyl) or U_iso_(H) = 1.2U_eq_ (other). A racemic twin model has been used in the final refinement with twin ratio 0.65 (3):0.35.

### Crystal data

**Table:**

[Zn(C_5_H_7_O_2_)_2_(C_9_H_9_NO)]	*F*(000) = 856
*M**_r_* = 410.77	*D*_x_ = 1.430 Mg m^−^^3^
Orthorhombic, *P*2_1_2_1_2_1_	Cu *K*α radiation, λ = 1.54180 Å
Hall symbol: P 2ac 2ab	Cell parameters from 1999 reflections
*a* = 9.5009 (3) Å	θ = 4.4–69.6°
*b* = 14.1674 (4) Å	µ = 2.03 mm^−^^1^
*c* = 14.2407 (5) Å	*T* = 200 K
*V* = 1916.84 (11) Å^3^	Blocks, white
*Z* = 4	0.28 × 0.15 × 0.08 mm

### Data collection

**Table:**

Nonius KappaCCD diffractometer	3600 independent reflections
Radiation source: fine-focus sealed tube	3466 reflections with *I* > 2σ(*I*)
horizontally mounted graphite crystal	*R*_int_ = 0.035
Detector resolution: 9 pixels mm^-1^	θ_max_ = 69.6°, θ_min_ = 4.4°
φ and ω scans	*h* = −11→11
Absorption correction: part of the refinement model (Δ*F*) (Parkin *et al.*, 1995)	*k* = −17→17
*T*_min_ = 0.542, *T*_max_ = 0.859	*l* = −17→17
8596 measured reflections	

### Refinement

**Table:**

Refinement on *F*^2^	Primary atom site location: structure-invariant direct methods
Least-squares matrix: full	Secondary atom site location: difference Fourier map
*R*[*F*^2^ > 2σ(*F*^2^)] = 0.030	Hydrogen site location: inferred from neighbouring sites
*wR*(*F*^2^) = 0.080	H-atom parameters constrained
*S* = 1.05	*w* = 1/[σ^2^(*F*_o_^2^) + (0.0434*P*)^2^ + 0.5913*P*] where *P* = (*F*_o_^2^ + 2*F*_c_^2^)/3
3600 reflections	(Δ/σ)_max_ < 0.001
236 parameters	Δρ_max_ = 0.24 e Å^−^^3^
0 restraints	Δρ_min_ = −0.29 e Å^−^^3^
0 constraints	

### Special details

**Table:**

Experimental. Absorption correction: Parkin *et al.*, 1995. Cubic fit to sin(θ)/λ; 24 parameters ^1^H NMR [300 MHz: CDCl_3_]: δ_H_ (*vs*. TMS) = 1.87 [s, 12H, –C*H*_3_], 4.03 [t, *J *= 11.7 Hz, 2H, –C*H*_2_N], 4.48 [t, 2H, –C*H*_2_O], 5.26 [s, 2H, –C*H*], 7.38 [m, 4H, Ar*H*], 7.85 [d, *J* = 8.8 Hz,1*H*, Ar*H*]; ^13^C{^1^H}NMR (75 MHz; CDCl_3_): δ_C_ (*vs*. TMS) = 28.1, 53.7, 68.2, 99.9, 125.9, 128.2, 129.0, 132.0, 165.0(w), 193.1).
Geometry. All e.s.d.'s (except the e.s.d. in the dihedral angle between two l.s. planes) are estimated using the full covariance matrix. The cell e.s.d.'s are taken into account individually in the estimation of e.s.d.'s in distances, angles and torsion angles; correlations between e.s.d.'s in cell parameters are only used when they are defined by crystal symmetry. An approximate (isotropic) treatment of cell e.s.d.'s is used for estimating e.s.d.'s involving l.s. planes.
Refinement. Refinement of *F*^2^ against ALL reflections. The weighted *R*-factor *wR* and goodness of fit *S* are based on *F*^2^, conventional *R*-factors *R* are based on *F*, with *F* set to zero for negative *F*^2^. The threshold expression of *F*^2^ > σ(*F*^2^) is used only for calculating *R*-factors(gt) *etc*. and is not relevant to the choice of reflections for refinement. *R*-factors based on *F*^2^ are statistically about twice as large as those based on *F*, and *R*- factors based on ALL data will be even larger.

### Fractional atomic coordinates and isotropic or equivalent isotropic displacement parameters (Å^2^)

**Table:**

	*x*	*y*	*z*	*U*_iso_*/*U*_eq_	
C1	0.3061 (3)	−0.0086 (3)	−0.06798 (17)	0.0483 (7)	
H1A	0.2514	0.0460	−0.0870	0.058*	
H1B	0.2575	−0.0654	−0.0879	0.058*	
C2	0.4526 (3)	−0.0046 (2)	−0.10858 (16)	0.0433 (6)	
H2A	0.4650	−0.0522	−0.1569	0.052*	
H2B	0.4720	0.0570	−0.1352	0.052*	
C3	0.4621 (2)	−0.01785 (18)	0.04907 (15)	0.0316 (5)	
C4	0.5407 (3)	−0.02065 (17)	0.13820 (16)	0.0319 (5)	
C5	0.6862 (3)	−0.01280 (19)	0.13607 (17)	0.0374 (5)	
H5	0.7331	−0.0086	0.0789	0.045*	
C6	0.7613 (3)	−0.0112 (2)	0.21951 (18)	0.0430 (6)	
H6	0.8589	−0.0061	0.2181	0.052*	
C7	0.6925 (3)	−0.0173 (2)	0.30468 (18)	0.0406 (6)	
H7	0.7435	−0.0149	0.3604	0.049*	
C8	0.5487 (3)	−0.0269 (2)	0.30720 (18)	0.0418 (6)	
H8	0.5027	−0.0317	0.3646	0.050*	
C9	0.4715 (3)	−0.02941 (19)	0.22414 (18)	0.0378 (6)	
H9	0.3743	−0.0369	0.2260	0.045*	
C10	−0.1142 (4)	−0.2000 (3)	−0.0308 (2)	0.0620 (9)	
H10A	−0.1390	−0.1528	−0.0763	0.093*	
H10B	−0.1980	−0.2232	−0.0010	0.093*	
H10C	−0.0667	−0.2512	−0.0616	0.093*	
C11	−0.0186 (3)	−0.1573 (2)	0.0420 (2)	0.0426 (6)	
C12	0.0308 (3)	−0.21237 (19)	0.1148 (2)	0.0485 (7)	
H12	0.0107	−0.2766	0.1119	0.058*	
C13	0.1077 (3)	−0.18128 (18)	0.1921 (2)	0.0409 (6)	
C14	0.1554 (4)	−0.2508 (2)	0.2652 (2)	0.0602 (8)	
H14A	0.2077	−0.2183	0.3129	0.090*	
H14B	0.2141	−0.2977	0.2364	0.090*	
H14C	0.0748	−0.2807	0.2929	0.090*	
C15	0.0715 (4)	0.2718 (2)	−0.0305 (2)	0.0611 (9)	
H15A	0.0125	0.2405	−0.0756	0.092*	
H15B	0.1545	0.2951	−0.0612	0.092*	
H15C	0.0210	0.3235	−0.0030	0.092*	
C16	0.1131 (3)	0.20281 (19)	0.0454 (2)	0.0430 (7)	
C17	0.1982 (3)	0.23396 (18)	0.1187 (2)	0.0466 (7)	
H17	0.2349	0.2946	0.1139	0.056*	
C18	0.2333 (3)	0.18219 (18)	0.19881 (19)	0.0407 (6)	
C19	0.3242 (4)	0.2251 (2)	0.2731 (2)	0.0591 (9)	
H19A	0.3384	0.1802	0.3228	0.089*	
H19B	0.2791	0.2804	0.2978	0.089*	
H19C	0.4135	0.2421	0.2466	0.089*	
N1	0.32955 (19)	−0.00883 (16)	0.03537 (13)	0.0343 (4)	
O1	0.54378 (17)	−0.02335 (15)	−0.02807 (12)	0.0415 (4)	
O2	0.0063 (2)	−0.06991 (14)	0.03232 (13)	0.0445 (5)	
O3	0.1428 (2)	−0.09589 (12)	0.20636 (12)	0.0397 (4)	
O4	0.0640 (2)	0.12044 (14)	0.03780 (13)	0.0454 (5)	
O5	0.1921 (2)	0.09787 (12)	0.21440 (13)	0.0402 (4)	
Zn1	0.14228 (3)	0.00792 (2)	0.10978 (2)	0.03289 (11)	

### Atomic displacement parameters (Å^2^)

**Table:**

	*U*^11^	*U*^22^	*U*^33^	*U*^12^	*U*^13^	*U*^23^
C1	0.0386 (13)	0.078 (2)	0.0279 (12)	−0.0026 (15)	−0.0031 (10)	0.0011 (15)
C2	0.0376 (12)	0.0633 (17)	0.0291 (11)	0.0052 (13)	−0.0037 (9)	0.0008 (15)
C3	0.0341 (12)	0.0299 (13)	0.0307 (11)	−0.0014 (10)	0.0023 (9)	−0.0004 (10)
C4	0.0354 (12)	0.0272 (12)	0.0331 (11)	0.0032 (10)	−0.0031 (9)	−0.0007 (9)
C5	0.0336 (12)	0.0412 (14)	0.0374 (12)	0.0001 (11)	0.0012 (9)	0.0030 (11)
C6	0.0332 (12)	0.0524 (17)	0.0435 (14)	−0.0003 (13)	−0.0040 (10)	0.0027 (14)
C7	0.0400 (13)	0.0421 (14)	0.0398 (13)	0.0048 (12)	−0.0103 (10)	−0.0032 (12)
C8	0.0427 (14)	0.0507 (17)	0.0321 (12)	0.0070 (12)	−0.0009 (10)	0.0006 (11)
C9	0.0313 (12)	0.0457 (16)	0.0365 (13)	0.0020 (11)	0.0007 (10)	−0.0022 (11)
C10	0.068 (2)	0.0600 (19)	0.0585 (19)	−0.0184 (17)	−0.0087 (16)	−0.0061 (16)
C11	0.0338 (14)	0.0421 (15)	0.0519 (16)	−0.0045 (12)	0.0040 (12)	−0.0090 (12)
C12	0.0532 (17)	0.0314 (13)	0.0610 (18)	−0.0033 (12)	−0.0049 (15)	−0.0036 (14)
C13	0.0409 (15)	0.0313 (13)	0.0505 (15)	0.0014 (11)	0.0046 (12)	0.0035 (11)
C14	0.067 (2)	0.0442 (16)	0.069 (2)	0.0031 (16)	−0.0091 (18)	0.0123 (15)
C15	0.080 (2)	0.049 (2)	0.0546 (19)	0.0087 (17)	−0.0094 (18)	0.0076 (15)
C16	0.0487 (17)	0.0333 (14)	0.0471 (16)	0.0081 (12)	0.0045 (13)	0.0025 (11)
C17	0.0545 (16)	0.0306 (13)	0.0548 (18)	−0.0056 (12)	0.0001 (14)	0.0031 (13)
C18	0.0430 (15)	0.0346 (14)	0.0446 (15)	−0.0032 (12)	0.0031 (12)	−0.0030 (11)
C19	0.073 (2)	0.0449 (17)	0.0589 (19)	−0.0137 (16)	−0.0123 (16)	−0.0033 (14)
N1	0.0333 (10)	0.0416 (11)	0.0281 (9)	−0.0003 (10)	−0.0020 (7)	−0.0012 (9)
O1	0.0335 (8)	0.0607 (13)	0.0303 (8)	0.0036 (9)	0.0014 (7)	0.0009 (8)
O2	0.0448 (11)	0.0447 (12)	0.0439 (10)	−0.0115 (9)	−0.0053 (8)	0.0031 (9)
O3	0.0431 (10)	0.0378 (9)	0.0384 (9)	−0.0065 (9)	0.0006 (9)	0.0031 (7)
O4	0.0506 (12)	0.0378 (10)	0.0478 (11)	0.0042 (9)	−0.0092 (9)	0.0004 (8)
O5	0.0517 (12)	0.0340 (9)	0.0350 (10)	−0.0027 (8)	0.0014 (8)	−0.0017 (7)
Zn1	0.03214 (17)	0.03310 (17)	0.03342 (17)	−0.00265 (14)	0.00149 (12)	0.00024 (14)

### Geometric parameters (Å, °)

**Table:**

C1—N1	1.489 (3)	C11—C12	1.379 (4)
C1—C2	1.508 (3)	C12—C13	1.393 (4)
C1—H1A	0.9700	C12—H12	0.9300
C1—H1B	0.9700	C13—O3	1.271 (3)
C2—O1	1.461 (3)	C13—C14	1.503 (4)
C2—H2A	0.9700	C14—H14A	0.9600
C2—H2B	0.9700	C14—H14B	0.9600
C3—N1	1.281 (3)	C14—H14C	0.9600
C3—O1	1.347 (3)	C15—C16	1.510 (4)
C3—C4	1.473 (3)	C15—H15A	0.9600
C4—C5	1.387 (4)	C15—H15B	0.9600
C4—C9	1.395 (4)	C15—H15C	0.9600
C5—C6	1.387 (3)	C16—O4	1.261 (4)
C5—H5	0.9300	C16—C17	1.393 (4)
C6—C7	1.381 (4)	C17—C18	1.397 (4)
C6—H6	0.9300	C17—H17	0.9300
C7—C8	1.373 (4)	C18—O5	1.277 (3)
C7—H7	0.9300	C18—C19	1.495 (4)
C8—C9	1.392 (4)	C19—H19A	0.9600
C8—H8	0.9300	C19—H19B	0.9600
C9—H9	0.9300	C19—H19C	0.9600
C10—C11	1.506 (4)	N1—Zn1	2.0844 (19)
C10—H10A	0.9600	O2—Zn1	2.0253 (19)
C10—H10B	0.9600	O3—Zn1	2.0136 (17)
C10—H10C	0.9600	O4—Zn1	2.0359 (19)
C11—O2	1.268 (4)	O5—Zn1	2.0169 (18)
			
N1—C1—C2	103.94 (19)	C12—C13—C14	119.9 (3)
N1—C1—H1A	111.0	C13—C14—H14A	109.5
C2—C1—H1A	111.0	C13—C14—H14B	109.5
N1—C1—H1B	111.0	H14A—C14—H14B	109.5
C2—C1—H1B	111.0	C13—C14—H14C	109.5
H1A—C1—H1B	109.0	H14A—C14—H14C	109.5
O1—C2—C1	103.86 (18)	H14B—C14—H14C	109.5
O1—C2—H2A	111.0	C16—C15—H15A	109.5
C1—C2—H2A	111.0	C16—C15—H15B	109.5
O1—C2—H2B	111.0	H15A—C15—H15B	109.5
C1—C2—H2B	111.0	C16—C15—H15C	109.5
H2A—C2—H2B	109.0	H15A—C15—H15C	109.5
N1—C3—O1	116.6 (2)	H15B—C15—H15C	109.5
N1—C3—C4	129.2 (2)	O4—C16—C17	124.9 (3)
O1—C3—C4	114.17 (19)	O4—C16—C15	116.2 (3)
C5—C4—C9	119.7 (2)	C17—C16—C15	118.9 (3)
C5—C4—C3	118.9 (2)	C16—C17—C18	125.8 (2)
C9—C4—C3	121.3 (2)	C16—C17—H17	117.1
C6—C5—C4	119.7 (2)	C18—C17—H17	117.1
C6—C5—H5	120.1	O5—C18—C17	124.1 (3)
C4—C5—H5	120.1	O5—C18—C19	115.8 (3)
C7—C6—C5	120.5 (2)	C17—C18—C19	120.2 (3)
C7—C6—H6	119.7	C18—C19—H19A	109.5
C5—C6—H6	119.7	C18—C19—H19B	109.5
C8—C7—C6	120.0 (2)	H19A—C19—H19B	109.5
C8—C7—H7	120.0	C18—C19—H19C	109.5
C6—C7—H7	120.0	H19A—C19—H19C	109.5
C7—C8—C9	120.3 (2)	H19B—C19—H19C	109.5
C7—C8—H8	119.9	C3—N1—C1	107.34 (19)
C9—C8—H8	119.9	C3—N1—Zn1	140.65 (16)
C8—C9—C4	119.7 (2)	C1—N1—Zn1	112.00 (14)
C8—C9—H9	120.2	C3—O1—C2	106.74 (17)
C4—C9—H9	120.2	C11—O2—Zn1	126.22 (19)
C11—C10—H10A	109.5	C13—O3—Zn1	125.81 (17)
C11—C10—H10B	109.5	C16—O4—Zn1	123.14 (18)
H10A—C10—H10B	109.5	C18—O5—Zn1	122.33 (18)
C11—C10—H10C	109.5	O3—Zn1—O5	87.50 (7)
H10A—C10—H10C	109.5	O3—Zn1—O2	88.62 (8)
H10B—C10—H10C	109.5	O5—Zn1—O2	153.64 (8)
O2—C11—C12	124.8 (3)	O3—Zn1—O4	156.45 (8)
O2—C11—C10	115.4 (3)	O5—Zn1—O4	87.87 (8)
C12—C11—C10	119.7 (3)	O2—Zn1—O4	85.36 (8)
C11—C12—C13	126.4 (3)	O3—Zn1—N1	105.19 (8)
C11—C12—H12	116.8	O5—Zn1—N1	104.31 (8)
C13—C12—H12	116.8	O2—Zn1—N1	101.87 (8)
O3—C13—C12	124.4 (3)	O4—Zn1—N1	98.33 (8)
O3—C13—C14	115.7 (3)		
			
N1—C1—C2—O1	11.7 (3)	C10—C11—O2—Zn1	−174.4 (2)
N1—C3—C4—C5	−167.8 (3)	C12—C13—O3—Zn1	−16.7 (4)
O1—C3—C4—C5	10.4 (4)	C14—C13—O3—Zn1	163.0 (2)
N1—C3—C4—C9	11.2 (5)	C17—C16—O4—Zn1	−17.8 (4)
O1—C3—C4—C9	−170.6 (2)	C15—C16—O4—Zn1	163.8 (2)
C9—C4—C5—C6	−1.7 (4)	C17—C18—O5—Zn1	26.4 (4)
C3—C4—C5—C6	177.3 (2)	C19—C18—O5—Zn1	−153.8 (2)
C4—C5—C6—C7	−0.1 (5)	C13—O3—Zn1—O5	174.6 (2)
C5—C6—C7—C8	1.3 (5)	C13—O3—Zn1—O2	20.7 (2)
C6—C7—C8—C9	−0.7 (5)	C13—O3—Zn1—O4	95.8 (3)
C7—C8—C9—C4	−1.0 (4)	C13—O3—Zn1—N1	−81.2 (2)
C5—C4—C9—C8	2.2 (4)	C18—O5—Zn1—O3	167.7 (2)
C3—C4—C9—C8	−176.7 (2)	C18—O5—Zn1—O2	−110.5 (2)
O2—C11—C12—C13	4.3 (5)	C18—O5—Zn1—O4	−35.4 (2)
C10—C11—C12—C13	−173.1 (3)	C18—O5—Zn1—N1	62.7 (2)
C11—C12—C13—O3	0.4 (5)	C11—O2—Zn1—O3	−16.6 (2)
C11—C12—C13—C14	−179.3 (3)	C11—O2—Zn1—O5	−98.1 (3)
O4—C16—C17—C18	−5.6 (5)	C11—O2—Zn1—O4	−173.8 (2)
C15—C16—C17—C18	172.8 (3)	C11—O2—Zn1—N1	88.7 (2)
C16—C17—C18—O5	0.8 (5)	C16—O4—Zn1—O3	110.2 (3)
C16—C17—C18—C19	−179.0 (3)	C16—O4—Zn1—O5	31.4 (2)
O1—C3—N1—C1	0.8 (4)	C16—O4—Zn1—O2	−174.1 (2)
C4—C3—N1—C1	178.9 (3)	C16—O4—Zn1—N1	−72.7 (2)
O1—C3—N1—Zn1	−177.5 (2)	C3—N1—Zn1—O3	−51.4 (3)
C4—C3—N1—Zn1	0.6 (5)	C1—N1—Zn1—O3	130.4 (2)
C2—C1—N1—C3	−8.1 (4)	C3—N1—Zn1—O5	39.9 (3)
C2—C1—N1—Zn1	170.75 (19)	C1—N1—Zn1—O5	−138.3 (2)
N1—C3—O1—C2	7.2 (3)	C3—N1—Zn1—O2	−143.2 (3)
C4—C3—O1—C2	−171.2 (2)	C1—N1—Zn1—O2	38.5 (2)
C1—C2—O1—C3	−11.5 (3)	C3—N1—Zn1—O4	129.8 (3)
C12—C11—O2—Zn1	8.1 (4)	C1—N1—Zn1—O4	−48.4 (2)
